# A fully synthetic textual dataset of student learning habits and preferences generated using a large language model

**DOI:** 10.1016/j.dib.2026.112512

**Published:** 2026-01-28

**Authors:** Mehedi Hasan

**Affiliations:** Faculty of Computer Science and Engineering, Patuakhali Science and Technology University, Patuakhali 8602, Bangladesh

**Keywords:** Synthetic dataset, Student learning habits, Large language model, Natural language processing, Educational data mining, Machine learning

## Abstract

Educational data mining and learning analytics have become important research areas for supporting pedagogical analysis, algorithm development, and privacy-preserving educational research. The advancement of natural language processing (NLP) methods in educational contexts depends on the availability of structured and well-documented textual datasets; however, access to real student data is often restricted due to ethical, legal, and privacy concerns. This article presents a fully synthetic textual dataset of student learning habits and preferences generated using a large language model (LLM). The dataset contains 10,000 CSV-formatted records representing fictional students and includes attributes such as education level, study hours, preferred learning methods, learning challenges, motivation levels, opinions on online learning, and primary devices used for study. Data generation was performed using structured prompting strategies with explicitly defined controlled vocabularies to ensure internal consistency and reproducibility while avoiding the use of any real personal information. The resulting dataset follows intentionally controlled and near-uniform distributions, with variables generated under independent constraints. This design limits its suitability for modelling real-world stochastic behaviour or discovering natural correlations but makes it appropriate for benchmarking educational NLP pipelines, evaluating synthetic data generation techniques, and conducting privacy-preserving survey and machine learning experiments.

Specifications Table

**Table:** 

Subject	Computer Sciences
Specific subject area	Natural Language Processing, Educational Data Mining, Synthetic Data Generation, Machine Learning
Type of data	Raw: CSV
Data collection	A total of 10,000 synthetic records were generated in 2025 using the ChatGPT web interface (GPT-4.1, 2025 release). The generation process employed structured prompts with predefined constraints on vocabularies, ranges, and distributions to simulate realistic student responses. Records were organized into a single CSV file with columns for respondent ID, education level, study hours, preferred learning method, main challenge, motivation level, online learning opinion, and device used. The dataset is compatible with standard data analysis tools and machine learning frameworks, supporting tasks such as text classification, sentiment analysis, and clustering.
Data source location	The dataset was generated computationally at Patuakhali Science and Technology University (22.4667° N, 90.3833° E), Patuakhali, Bangladesh.
Data accessibility	Repository name: Mendeley Data Data identification number: 10.17632/fysyzdknsk.3 Direct URL to data: https://data.mendeley.com/datasets/fysyzdknsk/3
Related research article	None.

## 1 Value of the Data

**Fig. 1:**
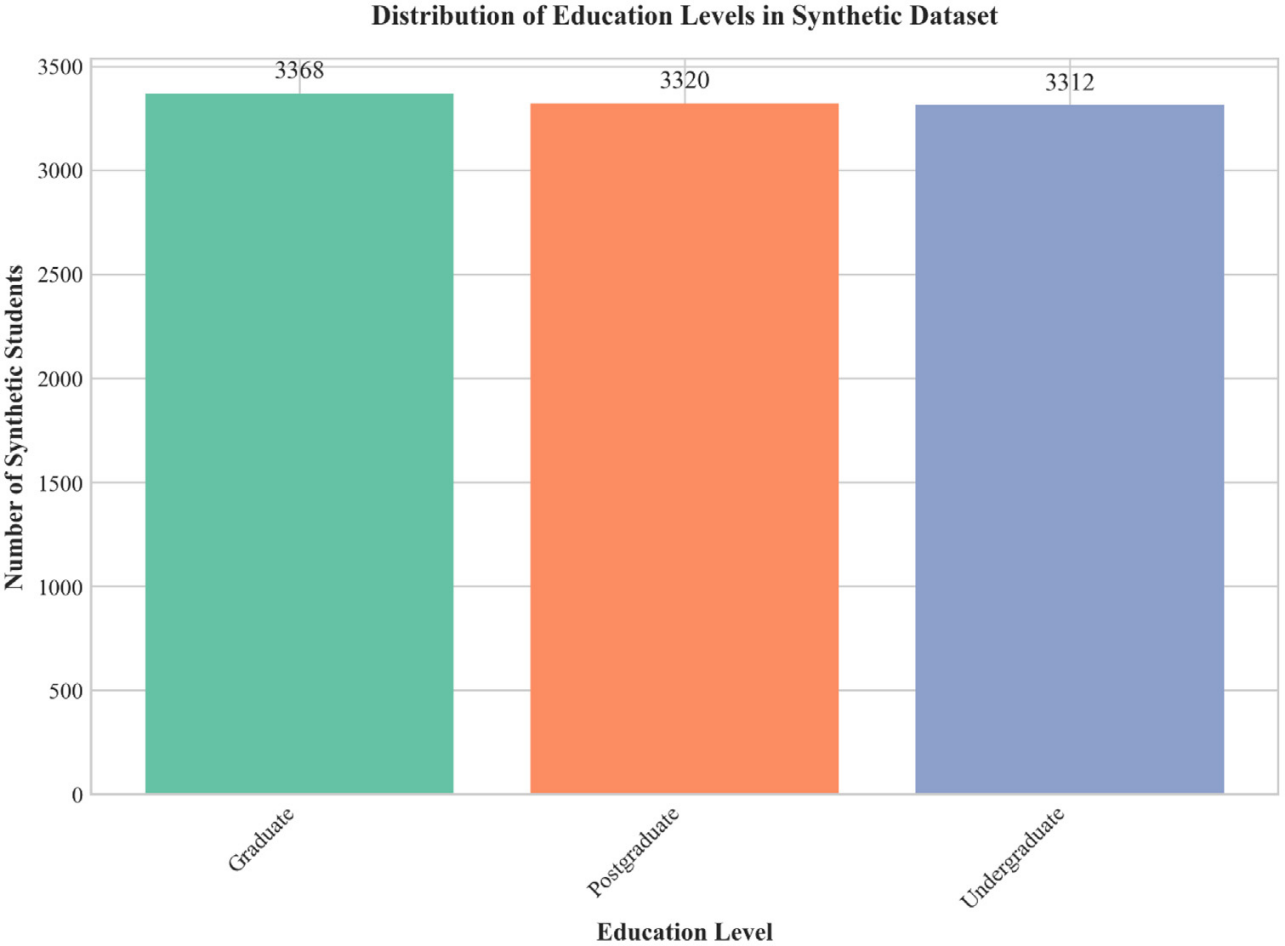
Distribution of education levels in the synthetic dataset, showing balanced representation across categories. Graduate (33.68 %), Postgraduate (33.20 %), and Undergraduate (33.12 %) students are nearly equally represented.
• All records are synthetically generated with controlled diversity, covering multiple education
  levels, challenges, and opinions. This supports educational researchers and NLP developers in tasks such as sentiment analysis, topic modelling, and predictive analytics in educational contexts. The relationship between study hours and education level (Fig. 2) demonstrates controlled synthetic patterns suitable for benchmarking.

The dataset comprises 10,000 fully synthetic records simulating student learning behaviors, generated in 2025. Its technical strengths are reflected in four areas:

• This dataset provides structured and textual data across various educational attributes,
  enabling researchers and practitioners in educational data mining, learning analytics, and natural language processing—as well as developers of educational technology—to train and evaluate machine learning models such as BERT, GPT variants, and scikit-learn classifiers with diverse, balanced samples. The balanced distribution across education levels is illustrated in Fig. 1.

**Fig. 2:**
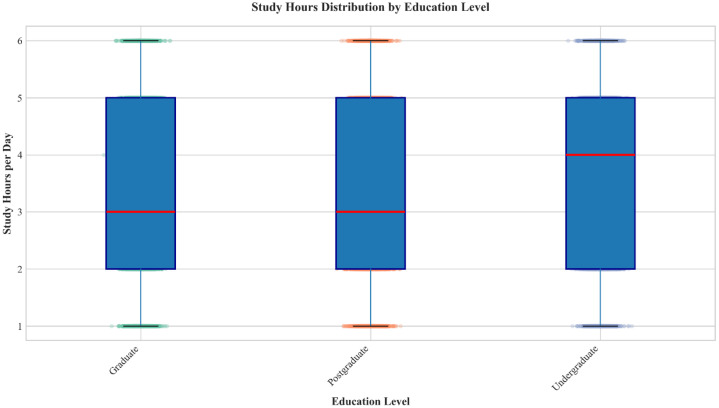
Box plot of study hours showing median study hours (3–4 h) and identical interquartile ranges (2–5 h) across all education levels. Individual data points indicate the distribution’s minimum (1 hour) and maximum (6 h) boundaries.
• The data mimics natural language variability in student responses, including free-text
  opinions, making it applicable for computational linguistics researchers and machine learning engineers developing robust NLP pipelines and benchmarking algorithms under privacy-safe conditions. The composite dashboard (Fig. 4) shows the distribution of motivation levels, device usage, and study habits.
• In addition to core attributes, the dataset includes metadata on generation parameters,
  allowing synthetic data researchers and ethics-focused computing teams to explore synthetic data quality, bias mitigation, and transfer learning across educational domains. The correlation matrix (Table 4) and heatmap (Fig. 3) provide quantitative insights into the dataset's variable relationships and associations.

**Fig. 3:**
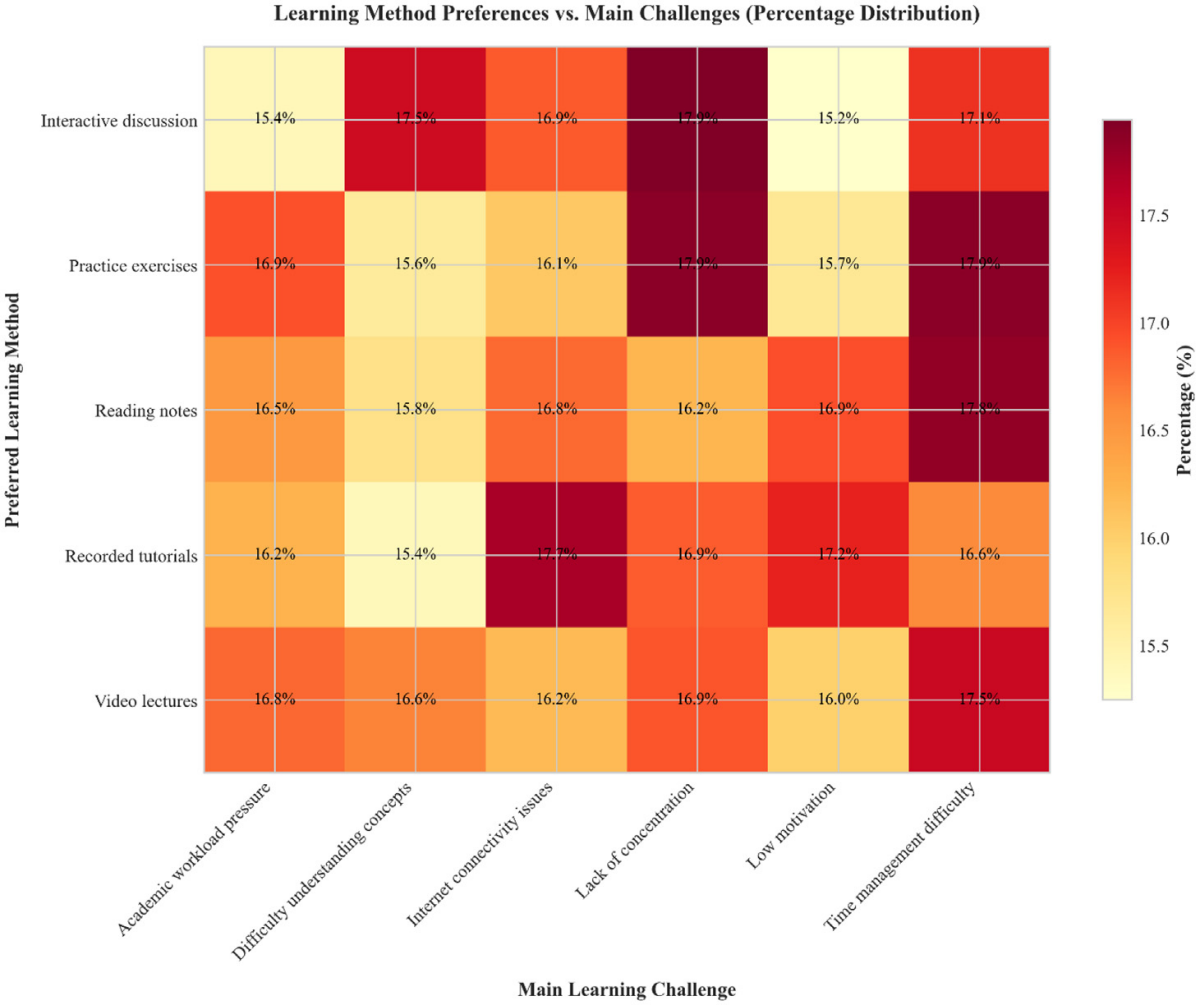
Heatmap showing the relationship between preferred learning methods and main learning challenges (percentage distribution).

## 2 Background

Online education expanded rapidly worldwide during the COVID-19 pandemic, with UNESCO reporting that >1.6 billion learners were affected by full or partial school closures, accelerating the adoption of digital learning platforms [1,2]. Understanding students’ learning habits, preferences, and challenges are essential for improving educational outcomes; however, the collection of real student data raises significant privacy and ethical concerns under regulations such as the General Data Protection Regulation (GDPR) and the Family Educational Rights and Privacy Act (FERPA). Synthetic data generation has emerged as a promising privacy-preserving solution, enabling the creation of realistic yet anonymized datasets suitable for research and analysis [3]. Recent advances in large language models (LLMs), particularly transformer-based architectures, have demonstrated strong capabilities in generating coherent and human-like text, supporting their use in data simulation and educational research contexts [4]. Existing educational datasets, such as the “Student Performance” dataset [5], are derived from real surveys and classroom records, which limits their scalability, availability, and reuse due to privacy constraints [6]. In parallel, many synthetic text datasets developed for natural language processing focus on general language understanding tasks rather than domain specific educational behaviors [7]. In contrast, the proposed dataset offers a fully synthetic and education-focused resource that captures diverse learning habits, enabling privacy-compliant research in educational data mining and AI-driven personalization.

## 3 Data Description

The dataset is distributed as a comprehensive package containing three files: the primary data file synthetic_student_learning_dataset_10,000.csv (containing 10,000 synthetic records, approximately 1.2 MB), a detailed README.md documentation file, and a data_dictionary.csv metadata file. Each record in the CSV file represents a fictional student profile with diverse educational backgrounds and opinions, all expressed in English with textual fields mimicking natural survey responses. The supplementary files provide essential documentation, usage guidelines, and column-level metadata to ensure reproducibility and facilitate research applications. Summary statistics for key categorical fields are provided in Table 1.

**Table 1:** Summary statistics for categorical fields.

Field	Categories (Count)	Total
Education Level	Graduate: 3368, Postgraduate: 3320, Undergraduate: 3312	10,000
Preferred Learning Method	Interactive discussion: 2040, Video lectures: 2001, Practice exercises: 1998, Reading notes: 1997, Recorded tutorials: 1964	10,000
Main Learning Challenge	Time management: 1738, Lack of concentration: 1716, Internet issues: 1672, Workload pressure: 1636, Low motivation: 1620, Concept difficulty: 1618	10,000
Motivation Level	Medium: 3364, Low: 3348, High: 3288	10,000
Device Used for Study	Tablet: 3425, Mobile: 3312, Laptop: 3263	10,000

The CSV file follows a standardized structure with the following columns, as detailed in Table 2. The dataset focuses exclusively on learning- behaviors and intentionally excludes demographic markers (see the EXPERIMENTAL DESIGN, MATERIALS AND METHODS section).

**Table 2:** Column descriptions.

Column Name	Description	Example	Controlled Vocabulary (Allowed Values)
respondent_id	Unique synthetic identifier (integer)	1	Not applicable (unique sequential identifier)
education_level	Academic level (categorical)	Undergraduate	Graduate, Postgraduate, Undergraduate
study_hours_per_day	Daily study hours (integer)	3	1, 2, 3, 4, 5, 6
preferred_learning_method	Preferred method of learning (categorical)	Video lectures	Interactive discussion, Practice exercises, Reading notes, Recorded tutorials, Video lectures
main_learning_challenge	Primary learning difficulty (categorical)	Time management difficulty	Academic workload pressure, Difficulty understanding concepts, Internet connectivity issues, Lack of concentration, Low motivation, Time management difficulty
motivation_level	Learning motivation level (categorical)	Medium	High, Low, Medium
online_learning_opinion	Opinion about online learning (free-text)	“I find online learning convenient but sometimes miss classroom interaction.”	Free text (10–15 words)
device_used_for_study	Primary study device (categorical)	Laptop	Laptop, Mobile, Tablet

To ensure reproducibility and clarify the boundaries of synthetic variation, all variables were generated using explicitly defined controlled vocabularies (see Appendix A for prompt templates). The complete set of allowed values for each field is provided in the data_dictionary.csv metadata file within the dataset package and is summarized in Table 2.

Table 3 summarizes descriptive statistics of study effort for the synthetic dataset and the UCI Student Performance dataset [5]. For the real-world dataset, the original ordinal 'studytime' variable (four weekly study-time categories: <2 h, 2–5 h, 5–10 h, >10 h) was mapped to approximate continuous values using representative midpoints (1.5, 3.5, 7.5, and 12.0 h per week), yielding a proxy variable (study_hours_approx) for distributional comparison. The synthetic dataset's daily study-hour attribute is presented alongside this proxy. Differences in measurement schema and granularity are noted; the comparison serves to familiarize the reader with the relative scale and spread of study time values across both datasets.

**Table 3:** Descriptive statistical comparison of study effort between the synthetic dataset and the UCI student performance dataset [5].

Metric	Synthetic Dataset (Study Hours per Day)	UCI Student Performance Dataset (Approx. Study Hours)
Mean	3.47	3.74
Standard Deviation	1.71	2.88
Minimum	1.00	1.00
Maximum	6.00	12.00
Median	3.00	3.50

A Spearman's rank correlation matrix was computed between study hours per day, education level, motivation level, and primary study device after ordinal encoding of categorical variables (Table 4). Correlations were close to zero across all variable pairs (absolute values ≤ 0.018), indicating the absence of nonsensical or unintended dependencies. This quantitative evidence validates the dataset's design as a controlled benchmark with independent variables, rather than as a statistical proxy for observational data.

**Table 4:** Spearman correlation matrix among key variables in the synthetic student learning dataset.

Variable	study_hours_per_day	education_level	motivation_level	device_used_for_study
study_hours_per_day	1.000	−0.003	−0.006	−0.017
education_level	−0.003	1.000	−0.007	−0.018
motivation_level	−0.006	−0.007	1.000	−0.001
device_used_for_study	−0.017	−0.018	−0.001	1.000

**Fig. 4:**
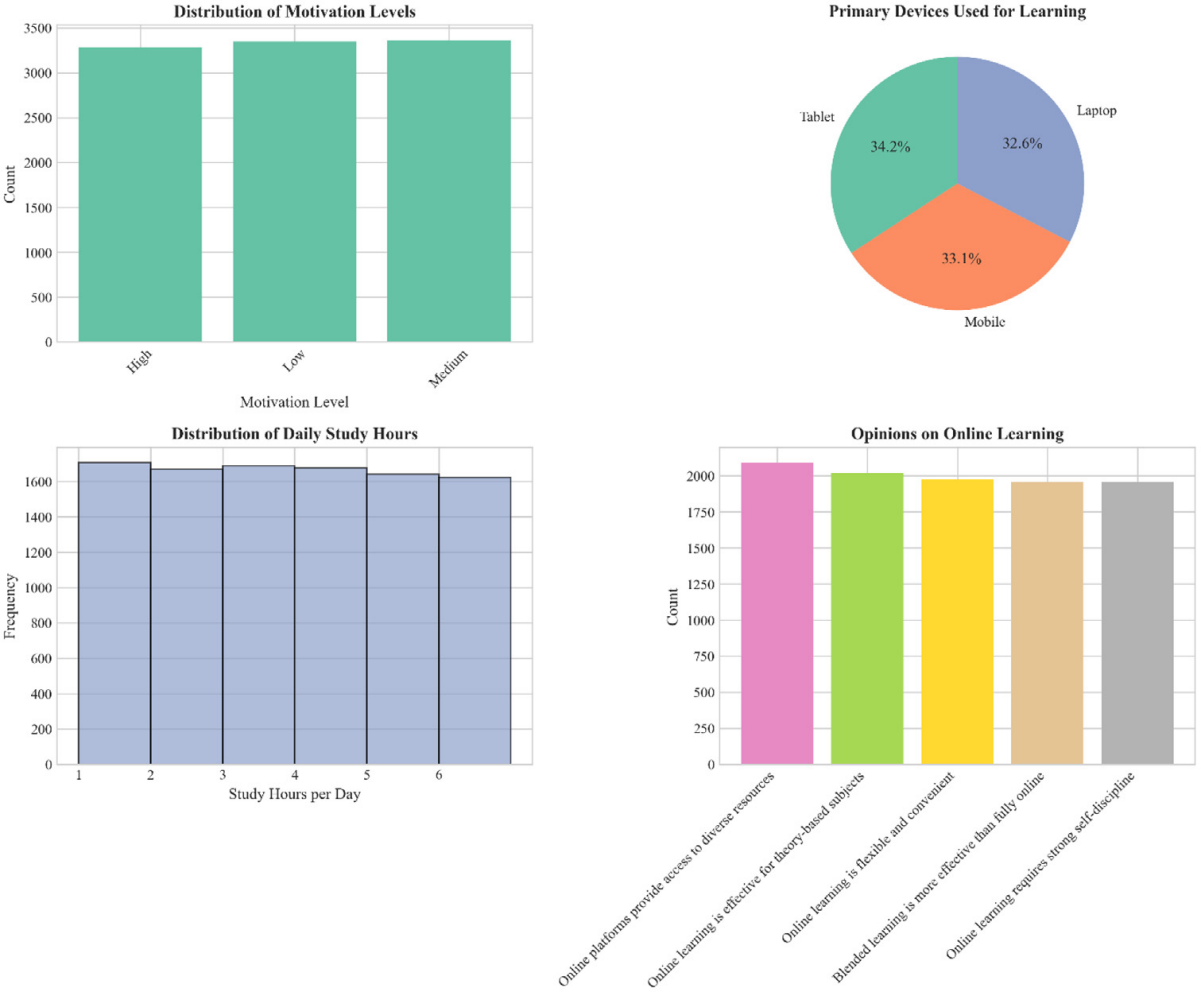
Composite dashboard showing additional dataset characteristics: (a)Motivation level distribution, (b) Device usage distribution, (c) Study hours histogram, and (d) Online learning opinion distribution (balanced across categories).

Visualizations of key dataset characteristics are presented in Fig. 1, Fig. 2, Fig. 3, Fig. 4. Fig. 1 shows the distribution of education levels across the synthetic dataset, illustrating balanced representation with approximately 33.3 % each for Graduate, Postgraduate, and Undergraduate students. Fig. 2 presents study hour patterns by education level, revealing that all groups share a similar interquartile range of 2 to 5 h. Undergraduate students exhibit a higher median study time (4 h) compared to Graduate and Postgraduate students (3 h). The distribution is uniform across all levels, with synthetic data points bounded strictly between 1 and 6 h.

Fig. 3 presents a heatmap visualization of the relationship between preferred learning methods and main learning challenges. The synthetic data reveals relatively uniform distribution across all categories, with percentages ranging from approximately 15 % to 18 %. Notable peaks include students who prefer recorded tutorials reporting internet connectivity issues (17.7 %) and those preferring practice exercises citing a lack of concentration (17.9 %). These distributions demonstrate that the synthetic dataset provides a balanced representation of common educational hurdles across various learning modalities. Fig. 4 provides a comprehensive dashboard of additional dataset characteristics. The composite visualization includes: (a) motivation level distribution, which is balanced across Low, Medium, and High categories; (b) primary device usage, showing a nearly equal preference for Tablets (34.2 %), Mobile devices (33.1 %), and Laptops (32.6 %); (c) a study hours histogram exhibiting a uniform distribution across the 1 to 6-hour range; and (d) online learning opinions, which are distributed evenly across various student perspectives. This dashboard format efficiently communicates multiple aspects of the dataset’s composition and demonstrates its suitability for various analytical applications.

## 4 Experimental Design, Materials and Methods

The dataset was generated using the ChatGPT web interface (GPT-4.1, 2025 release). Model parameters such as temperature and top-p were not user-configurable in the web interface and therefore remained at system-default settings. Data generation did not rely on automated API calls or scripting; instead, all records were produced through iterative structured prompting conducted manually via the chat interface. The exact prompt templates and controlled vocabularies used to generate the synthetic CSV records are provided in Appendix A to ensure transparency and reproducibility.

Prompts were explicitly designed to enforce predefined constraints across both categorical and numerical attributes. Education levels were generated with proportional representation (approximately 33.3 % each for undergraduate, graduate, and postgraduate students), study hours were constrained to integer values uniformly distributed between 1 and 6 h, and all categorical variables were restricted to explicitly defined controlled vocabularies to ensure consistency and standardization.

Free-text opinion fields were generated by instructing the model to produce varied and contextually coherent paragraphs conditioned on the structured attributes. To enhance diversity and reduce redundancy, records were generated in batches of approximately 1000 entries per prompting cycle.

All generated free-text opinion responses underwent post-generation screening to ensure the absence of toxic, offensive, or discriminatory language. This screening was conducted through a combination of prompt-level constraints—explicitly instructing the language model to avoid harmful, biased, or sensitive content—and manual inspection during dataset curation. Records containing inappropriate language, biased phrasing, semantically incoherent responses, or structural anomalies such as inconsistent text length or formatting errors were excluded. The dataset intentionally excludes explicit demographic identifiers such as gender, geographic location, ethnicity, or institution names. This design choice reduces the risk of reinforcing demographic stereotypes while maintaining focus on learning behaviour patterns.

To ensure balance and internal consistency, 12,000 synthetic records were initially generated, of which 2000 were discarded during manual curation. The resulting dataset comprises 10,000 validated records exhibiting controlled and near-uniform distributions across key variables. No real student data was used at any stage of the generation process, thereby preserving full synthetic integrity and eliminating privacy concerns. Table 5 summarizes the distribution of records across education levels before and after the manual anomaly removal process. This controlled generation and curation strategy supports the dataset’s intended use for educational NLP benchmarking, synthetic data evaluation, and reproducible machine learning experiments.

**Table 5:** Record count by education level before and after the manual cleaning process.

Education Level	Before Discarding Anomalies	After Discarding Anomalies (Current)
Graduate	4041	3368
Postgraduate	3984	3320
Undergraduate	3975	3312
Total	12,000	10,000

## Limitations

While this dataset offers a valuable synthetic resource for educational NLP research, it has limitations:

• Uniformity vs. Real-world Stochasticity: As an LLM-generated resource, the dataset exhibits highly controlled variability. The visualizations in Fig. 2, Fig. 4 demonstrate almost perfectly uniform distributions for study hours and motivation levels. While this ensures balance, it may not capture the “long-tail” distributions or the messy, non-linear patterns often found in real-world student behaviour.
• Independent Variable Generation & Controlled Correlation Structure: The correlation analysis (Table 4) quantitatively confirms the absence of strong variable relationships, which limits the dataset's utility for modelling real-world student behaviour but supports its purpose as a controlled benchmark.
• Absence of Domain-Specific Correlation: The heatmap in Fig. 3 reveals that the synthetic generation process produced a nearly even distribution across all learning challenges regardless of the preferred method. This lack of strong correlation (where all cells range between 15 % and 18 %) suggests that the model did not simulate specific pedagogical dependencies, such as “video learners” being more susceptible to “internet connectivity issues”.
• Narrow Distribution Range: The structured numerical data, such as daily study hours, is strictly bounded between 1 and 6 h. This excludes “extreme” learners (e.g., students
• studying 0 h or 10+ hours), which adds consistency but limits the dataset’s utility for outlier detection research.
• Device Representation: The device usage data (Fig. 4b) is evenly split between Tablets (34.2 %), Mobile (33.1 %), and Laptops (32.6 %), which may not reflect actual socioeconomic or regional trends where one device typically dominates the educational
• landscape.
• Modality Constraints: The dataset is currently limited to textual and structured numerical data; it lacks multimodal elements such as student-generated images, diagrams, or audio recordings.

Future research will focus on introducing natural stochasticity into data distributions to better reflect real-world “long-tail” student behaviours. Additionally, the generation pipeline will be refined to foster stronger pedagogical dependencies and incorporate multimodal elements like synthetic diagrams and longitudinal learning trajectories.

## Ethics Statement

This work does not involve human participants, animal experiments, or any personal or sensitive data. Therefore, no ethical approval was required for this study.

## CRediT Author Statement

**Mehedi Hasan:** Conceptualization, Methodology, Software, Validation, Formal analysis, Investigation, Resources, Data curation, Writing – Original Draft, Writing – Review & Editing, Visualization, Supervision, Project administration, Funding acquisition.

## Data Availability

Mendeley DataA Fully Synthetic Textual Dataset of Student Learning Habits and Preferences Generated Using a Large Language Model (Original data)

## Appendix Supplementary Materials

Image, application 1
