# Supplementary material for: A fully synthetic textual dataset of student learning habits and preferences generated using a large language model
Source: Data Brief. 2026 Jan 28;65:112512. doi: 10.1016/j.dib.2026.112512 (PMC12925479; doi:10.1016/j.dib.2026.112512)
Supplement: Supplementary file 1 [file mmc1.docx]

# Appendix A. Prompt Templates Used for Synthetic Data Generation

**Used Prompt**

You are a synthetic data generation system. Your task is to generate fully fictional and synthetic student learning records. Do not use, reference, or infer any real individuals, real surveys, or real-world datasets. All outputs must be entirely synthetic and anonymized by design, and must not contain offensive, discriminatory, or biased content. Now, generate the synthetic student learning data according to the schema and constraints defined below. Each record represents a fictional student profile created solely for research and benchmarking purposes in educational data mining and natural language processing.

Schema & Controlled Vocabularies:

1. respondent_id: A unique integer identifier (sequential).
2. education_level: One of: Graduate, Postgraduate, Undergraduate.
3. study_hours_per_day: An integer value between 1 and 6 (inclusive).
4. preferred_learning_method: One of: Interactive discussion, Practice exercises, Reading notes, Recorded tutorials, Video lectures.
5. main_learning_challenge: One of: Academic workload pressure, Difficulty understanding concepts, Internet connectivity issues, Lack of concentration, Low motivation, Time management difficulty.
6. motivation_level: One of: High, Low, Medium.
7. online_learning_opinion: A coherent, contextually appropriate fictional sentence expressing an opinion about online learning. It should be 10–15 words in length and reflect the student's other attributes (e.g., education level, motivation). Ensure diverse phrasing and avoid repetition across records.
8. device_used_for_study: One of: Laptop, Mobile, Tablet.

Generation Rules:

- All values must strictly conform to the allowed vocabularies and ranges specified above.
- The education_level values should be generated with the aim of achieving a roughly balanced distribution across the three categories.
- The study_hours_per_day values should follow a near-uniform distribution across the integer range 1–6.
- Categorical variables (preferred_learning_method, main_learning_challenge, motivation_level, device_used_for_study) should be generated to ensure broad coverage and diversity across their respective allowed values.
- The online_learning_opinion must be a unique and grammatically correct. It should be conditioned on the other fields in the record to simulate a realistic response but must remain entirely fictional and generic.
- Do not include any personal identifiers, names, specific locations, university names, or any other real-world references.
- Output must be formatted as comma-separated values (CSV), with one complete record per line. Include a header line with the exact column names as listed in the schema.

Strictly follow the above schema and generation rules to produce exactly 1,000 synthetic records, and output the result as a downloadable CSV file suitable for direct use in research and benchmarking workflows.

**Guidelines for Using This Prompt**

- Platform: Use the ChatGPT web interface with the GPT-4.1 model.
- Batch Generation: Run the above prompt twelve times iteratively to generate approximately 12000 initial records. For the second through twelfth batches, replace the final instruction sentence of the prompt (i.e., *“Strictly follow the above schema and generation rules…”*) with the following text:
  “Strictly follow the above schema and generation rules to produce exactly 1,000 additional synthetic records, ensuring that all respondent_id values continue sequentially from the last identifier used in the previous batch and that no records or free-text opinions are duplicated across batches. Output the result as a downloadable CSV file suitable for direct use in research and benchmarking workflows.”
